# ERS Congress 2025: highlights from the Interstitial Lung Diseases Assembly

**DOI:** 10.1183/23120541.00344-2026

**Published:** 2026-07-06

**Authors:** Johad Khoury, Aycan Yüksel, Nikoleta Bizymi, Antonio Fabozzi, Marta Garcia-Moyano, Marlies Wijsenbeek, Michael Kreuter, Laura Fabbri

**Affiliations:** 1 Pulmonary, Critical Care and Sleep Medicine, Yale University, New Haven, CT, USA; 2 Pulmonary Division, Carmel Medical Center and EMMS Nazareth Hospitals, Haifa, Israel; 3 Department of Respiratory Medicine, TOBB Economics and Technology University, Ankara, Turkey; 4 Laboratory of Molecular and Cellular Pneumonology, School of Medicine, University of Crete, Heraklion, Greece; 5 Department of Nursing, School of Health Sciences, National and Kapodistrian University of Athens, Athens, Greece; 6 Fifth Department of Respiratory Medicine, Sotiria Thoracic Diseases General Hospital of Athens, Athens, Greece; 7 Department of Public Health and Infectious Diseases, Pulmonology Unit, Policlinico Umberto I, “Sapienza” University of Rome, Rome, Italy; 8 Unit for Interstitial Lung Diseases, Department of Respiratory Medicine, Cruces University Hospital, Barakaldo, Spain; 9 BioCruces Bizkaia Health Research Institute, Barakaldo, Spain; 10 Centre of Interstitial Lung Diseases, Erasmus MC, University Medical Centre, Rotterdam, The Netherlands; 11 Mainz Center for Pulmonary Medicine, Department of Pneumology, Mainz University Medical Center, Mainz, Germany; 12 Department of Pulmonary, Critical Care and Sleep Medicine, Marienhaus Clinic Mainz, Mainz, Germany; 13 National Heart and Lung Institute, Imperial College London, London, UK; 14 Department of Respiratory Medicine, Royal Brompton Hospital, London, UK

## Abstract

2025 was a great year for research into interstitial lung diseases (ILDs) with many breakthrough discoveries, and the ILD sessions at the 2025 European Respiratory Society (ERS) Congress in Amsterdam, the Netherlands, reflected this. Multiple positive clinical trials, major advances in artificial intelligence (AI), and promising basic and translational bench work were presented. Here we summarise the major advances made in:

## Shareable abstract

**A summary of the interstitial lung disease presentations and posters from #ERSCongress 2025** https://bit.ly/4tScT0p

2025 was a great year for research into interstitial lung diseases (ILDs) with many breakthrough discoveries, and the ILD sessions at the 2025 European Respiratory Society (ERS) Congress in Amsterdam, the Netherlands, reflected this. Multiple positive clinical trials, major advances in artificial intelligence (AI), and promising basic and translational bench work were presented. Here we summarise the major advances made in:

- Classification of ILDs
- Novel treatments and management of idiopathic pulmonary fibrosis (IPF) and fibrotic lung diseases
- Prognostication and management of connective tissue disease-related ILDs (CTD-ILDs)
- AI and machine learning in ILD research

In the recently released joint ERS/American Thoracic Society update statement on interstitial pneumonias, several major conceptual and structural revisions were introduced (table 1). Classification now extends beyond idiopathic interstitial pneumonias alone, providing a broader framework applicable across ILDs. The updated system is organised into three overarching categories: interstitial patterns, alveolar filling patterns, and unclassifiable patterns. Among the interstitial patterns, the main subcategories are usual interstitial pneumonia (UIP), nonspecific interstitial pneumonia, and bronchiolocentric interstitial pneumonia, with the latter being a point of ongoing discussions. The alveolar filling category encompasses disorders such as organising pneumonia, respiratory bronchiolitis-associated ILD, and alveolar macrophage pneumonia. In addition, several entities have undergone changes in nomenclature to better reflect underlying pathology and disease behaviour, as summarised in table 1. One of the central aims of this update is to ensure that secondary causes are systematically considered and not overlooked during classification and diagnosis [1].

**TABLE 1:** Updated classification of interstitial pneumonias

New terminology (2025)	Previous terminology (2013)	Rationale
Idiopathic diffuse alveolar damage	Acute interstitial pneumonia	Other interstitial pneumonias can present acutely
Alveolar macrophage pneumonia	Desquamative interstitial pneumonia	The main pathological finding is macrophage infiltration
Bronchiolocentric interstitial pneumonia	Typical hypersensitivity pneumonia pattern	HP is an airway-centred disease Typical HP pattern is also associated with CTDs (22%), and 34% of HP is idiopathic

HP: hypersensitivity pneumonia; CTDs: connective tissue diseases.

A hot topic discussed at the 2025 ERS Congress was upfront combination of antifibrotic therapy for fibrosing ILDs. Treatment of deteriorating IPF or progressive fibrotic ILDs despite antifibrotic treatment is often challenging. Current evidence suggests continuation of the antifibrotic or switch to an alternative agent with potentially similar effect, and highlights the need for novel treatments [2, 3]. Add-on or upfront combination therapies may be on the horizon, as novel antifibrotics such as nerandomilast and treprostinil advance through clinical development [4, 5]. Although robust randomised trials for upfront combination regimens in newly diagnosed patients are lacking, first studies suggest potential additive effects. Earlier combination attempts, such as the INJOURNEY phase 2 trial, suggested potential additive effect [6], but were limited by short follow-up and tolerability concerns [7]. However, positive trials that were presented at the 2025 ERS Congress may have major impact on antifibrotic management in the near future: the phase 3 TETON-2 trial, reported efficacy of inhaled treprostinil in IPF, demonstrating reduced forced vital capacity (FVC) decline (−54 mL *versus* −107 mL), a 46% lower risk of acute exacerbations, and a 23% lower risk of mortality, with no new side effects or safety concerns reported [5]. However, full publication and confirmation in TETON-1 and TETON-PPF are pending. In parallel, long-term data from the FIBRONEER-ILD trial showed that nerandomilast, a selective phosphodiesterase 4B (PDE4B) inhibitor, sustained attenuation of FVC decline over 76 weeks (−130 mL *versus* −230 mL with placebo), with favourable effects on respiratory-related hospitalisations, mortality and time to first exacerbation, and an additive effect over nintedanib (in both low and high dose of nerandomilast) and pirfenidone (only in high nerandomilast dose) [8]. These effects that are beyond the common endpoint of FVC decline, which focus on mortality, disease exacerbation and respiratory-related hospitalisation, may be the common standard outcomes in future trials in antifibrotic medications.

Overall, while upfront combination antifibrotic therapy may represent an attractive concept with plausible mechanistic synergy, the sequence and timing of the combination need further investigation.

Further early-stage positive clinical trials that hold promise for ILD management in the future include the phase 2a AIR trial, which evaluated buloxibutid, a selective angiotensin II type-2 receptor agonist, administered for 36 weeks *versus* control. Treated patients exhibited a mean FVC change of +23 mL, compared with −114 mL in controls, suggesting potential disease modification; a 52-week extension is underway. The authors suggested enhanced surfactant synthesis and blocking transforming growth factor-β (TGF-β)1 signalling as an antifibrotic pathways, and nitric oxide-mediated vascular remodelling [9]. Similarly, zelasudil showed a milder FVC decline compared to placebo in pulmonary fibrosis in a phase 2 trial (−50 mL *versus* −100 mL, respectively. Zelasudil inhibits ROCK2, a central pathway in fibrotic processes, including pulmonary fibrosis [10]. Moreover, the TNIK inhibitor rentosertib attenuated pulmonary fibrosis through TGF-β/YAP-TAZ pathways, leading to lower expression of the fibrosis-associated molecules MMP10, COL1A1, FAP and LTBP2. The treatment group gained a mean of +188 mL in FVC, compared to −20 mL in the placebo group after 12 weeks of treatment [11].

Together, these studies underscore a paradigm shift towards targeted, pathway-specific antifibrotic strategies that integrate vascular, epithelial, and immune-metabolic modulation, potentially expanding the therapeutic armamentarium beyond traditional TGF-β inhibition.

In sarcoidosis, one of the major trials presented at the 2025 ERS Congress was the phase 3 EFZO-FIT trial, comparing efzofitimod (3 or 5 mg·kg^−1^) to placebo. The study included 268 patients, and over 48 weeks, the primary end point of corticosteroid dose reduction was not met, with treatment and placebo groups receiving comparable doses (p>0.05). However, 5 mg·kg^−1^ improved fatigue (fatigue assessment score; p=0.0226) and quality of life (King's Sarcoidosis Questionnaire – General Health; p=0.0197), with a higher proportion achieving ≥6 months steroid-free status and preserved FVC. Safety was comparable to placebo [12]. Furthermore, Maione *et al*. [13] (Odivelas, Portugal) demonstrated that infliximab achieved clinical response in 85% of pulmonary, 100% of cardiac, and 100% of neurosarcoidosis cases, although relapse rates were high upon withdrawal. Merenkova *et al*. [14] (Kyiv, Ukraine) reported that methotrexate plus hydroxychloroquine induced clinical remission in 62.5% and stabilisation in 25% of refractory sarcoidosis after 6 months of treatment, albeit with 12.5% discontinuations due to adverse events, emphasising the need for careful monitoring.

Moreover, significant efforts were made to optimise management and monitoring of hypersensitivity pneumonitis. It was reported that bronchoalveolar lavage lymphocytosis was significantly more frequent in progressors, suggesting persistent inflammation contributes to fibrosis even in chronic stages [15], and correlated with 1-year declines in FVC and diffusing capacity of the lung for carbon monoxide [16]. Furthermore, it was highlighted that peripheral eosinophilia (>500 per µL) was a strong predictor of progressive fibrotic hypersensitivity pneumonitis, proposing a potential immunological phenotype of disease acceleration [17].

Progress was also made in risk stratification and prognostication of CTD-ILD: the updated ERS and European Alliance of Associations for Rheumatology guidelines on CTD-ILD were also presented. The guidelines provide evidence-based recommendations for screening, diagnosis, monitoring, and treatment across major CTDs, including systemic sclerosis, rheumatoid arthritis, idiopathic inflammatory myopathies, Sjögren disease, systemic lupus erythematosus, and mixed CTD. The guidelines emphasise multidisciplinary evaluation, recommend baseline high-resolution computed tomography (HRCT) for screening in systemic sclerosis, and propose algorithms for ongoing monitoring and risk assessment. The guidelines highlight the low certainty of evidence for most therapies and encourage individualised management and further research to address knowledge gaps. In several CTDs and clinical scenarios, the task force found insufficient evidence to support specific recommendations, underscoring the need for ongoing investigation and multidisciplinary care.

Furthermore, A.M. Hoffmann-Vold (Oslo, Norway) presented a composite risk model for early ILD in Sjögren syndrome, incorporating age, erythrocyte sedimentation rate, and complement C3 levels, achieving an area under the curve (AUC) of 0.80 for ILD detection on HRCT [18]. Importantly, in the RECITAL cohort, rituximab efficacy was linked to the degree of B-cell depletion: patients achieving <5 B-cells per µL (in the B-cell depleted group) showed a mean FVC gain of +260 mL at 48 weeks, compared to +35 mL in non-depleted patients, supporting B-cell kinetics as a therapeutic biomarker [19].

Although it has been neglected until recently, non-pharmacological treatment and symptom relief were in the spotlight of major studies. A randomised trial demonstrated that sustained adherence (>80%) to a 5-year pulmonary rehabilitation (PR) programme was associated with a 45% reduction in mortality among ILD patients [20], while others showed that PR significantly improves 6-min walking distance and quality of life estimated by St George's Respiratory Questionnaire [21]. To increase accessibility and compliance to PR, the modern online patient education and self-management programme FILIP was developed; co-designed with patients, it provides evidence-based information and practical self-management strategies tailored for individuals with pulmonary fibrosis [22]. Moreover, symptom control rather than solely disease modulation is taking a significant part of the modern management of ILD. The phase 2 IPF-COMFORT study evaluated Orvepitant, a selective neurokinin-1 receptor antagonist targeting the hypersensitised cough reflex typical of IPF, and demonstrated significant cough relief [23]. Similarly, nalbuphine, a κ-opioid receptor agonist and μ-opioid receptor antagonist, was shown to reduce both daytime and nocturnal cough frequency in patients with IPF in a recent phase 2a trial [24].

Along with clinical development and the positive trials, significant scientific progress and early-phase trials were presented. Promising results have emerged from *in vitro* and mouse model studies using the small molecule AZD8965 to inhibit arginase, a key enzyme in the urea cycle, as a potential therapeutic target in pulmonary fibrosis [25]. Furthermore, to improve drug delivery and decrease side effects, Esposito *et al.* [26] showed compelling preclinical findings using inhaled small interfering RNA (siRNA) to knockdown the CD105 to block the TGF-β/SMAD pathway. Similarly, as part of the state of the art session “Horizon scanning in the treatment of interstitial lung diseases”, R. Chambers (London, UK) described how the inhaled siRNA TKR-250 targeting TGF-β1 phase 1 study was recently completed, showing favourable safety, and the inhaled ALK5 inhibitor AGMB-447 phase 1 study, and inhaled pirfenidone phase 2b trials are ongoing.

As AI exponentially advances, its use in pulmonology in general and ILD in particular is accelerating. Adegunsoye *et al*. [27] showed that the use of an AI algorithm with CT scans can detect progressive fibrosing ILD 21 months earlier than standard care. Another model identified reticulations and honeycomb cysts in systemic sclerosis ILD, and detected progression (AUC 0.8, positive predictive value 0.68 and negative predictive value 0.89) [28], while measuring airway and vessel volume predicted mortality in patients with CTD-ILD [29]. A combination of biological and radiological markers became more available thanks to the development of machine learning. Based on a combination of proteomic markers and ground-glass opacities, a precise prediction of 12-month progression was made [30]. Moreover, electronic nose (eNose) breath analysis could distinguish between UIP and non-UIP patterns (AUC 0.81, 95% CI 0.74–0.88), and within the patients that had a UIP pattern, IPF could be distinguished from non-IPF with an AUC of 0.91 (95% CI 0.79–1.00) [31].

The role of exposure in the development of ILD and related comorbidities cannot be overemphasised. This was discussed in the context of the EXPLAIN-IT inter-assembly Clinical Research Collaboration. Patients suffering from any type of ILD should not be considered in isolation from their environmental history, as identifying treatable traits through exposure profiling may improve outcomes, especially when combined with preventive strategies [32]. The field of exposomics can help clinicians and researchers better understand, manage, and prevent these devastating diseases [33]. Byssinosis, an ILD associated with a specific environmental causative agent, is considered an important health complication in workers exposed to cotton wool. Exposure-reduction strategies are crucial, as shown by occupational interventional trials, such as the MultiTex trial by Nafees and co-workers [34–36]. This was a cluster randomised controlled trial involving 38 textile mills in Karachi, Pakistan, where workers and their managers were trained in good practices, including wet mopping, safe disposal of cotton dust, and use of face masks. The intervention successfully led to improvement of respiratory symptoms and lung function of textile workers in cotton mills.

Moreover, tuberculosis prevalence is highly associated with silicosis, showing increased numbers among small-scale miners due to silica exposures, as shown in systematic reviews and meta-analyses published by Howlett and co-workers [37–39] and Mbuya *et al*. [40]. The diagnosis of tuberculosis in this group of individuals faces a lot of challenges [41], but at the same time is crucial due to the high mortality of the condition among miners [42]. Thus, reduction in cumulative exposure, which is both feasible and effective, can lead to better health outcomes in these populations [40, 43].

5 years after the COVID-19 pandemic, post-COVID ILD remains one of the most complicated post-COVID sequelae. Post-COVID ILD, defined as ILD developing at least 12 weeks after acute COVID-19, presents with various radiological patterns, including ground-glass opacities, reticulation, fibrosis, and traction bronchiectasis, and remains challenging to diagnose and treat. Recently, rehabilitation interventions, including digital ones, have been trialled in patients suffering from persistent symptoms [44–47]. The single-blind randomised controlled trial PHOSP-R, which compared face-to-face and remote exercise-based rehabilitation interventions to usual care in patients with long COVID, showed improvement in short-term exercise capacity [48]. While direct data on the effect of rehabilitation in post-COVID ILD are currently missing, extrapolation from data on other ILDs and long COVID is promising [46, 47, 49].

Despite the significant advancement in ILD care and research, racial and ethnic disparities still affect the evaluation and management of ILD and contribute to variable outcomes across patient groups. For instance, a higher misclassification rate of non-white individuals is observed in clinical trials. This is usually due to the use of race-specific, rather than race-equal, pulmonary function tests, affecting the precise treatment and diagnosis of non-white patients. However, such disparities are addressable through evidence-based approaches, public informational campaigns, and governmental policies, to ensure equal and consistent access to healthcare [50, 51].

In conclusion, ILDs have been re-classified based on the underlying pathogenesis. Significant advances in IPF and progressive fibrosing ILD treatment have been made, including the introduction of nerandomilast and the possible extension of indications for treprostinil to include these conditions. Furthermore, AI tools hold the promise for improving care in ILD, as technologies enhance detection and standardise disease assessment. Despite that, the field remains complex and requires intensive research and technologies to overcome current challenges.

The rapid development of AI in medical imaging, particularly in ILD diagnosis and quantitative assessment, together with the integration of physiological, biological and radiological data into machine learning models, offers important opportunities to improve precision medicine approaches. Continued multidisciplinary collaboration, robust clinical trials and sustained research investment will be essential to address current knowledge gaps and move the field closer to transformative outcomes for patients.
